# Radio frequency measurements of tunnel couplings and singlet–triplet spin states in Si:P quantum dots

**DOI:** 10.1038/ncomms9848

**Published:** 2015-11-09

**Authors:** M. G. House, T. Kobayashi, B. Weber, S. J. Hile, T. F. Watson, J. van der Heijden, S. Rogge, M. Y. Simmons

**Affiliations:** 1 Centre of Excellence for Quantum Computation and Communication Technology, School of Physics, University of New South Wales, Sydney, New South Wales 2052, Australia

## Abstract

Spin states of the electrons and nuclei of phosphorus donors in silicon are strong candidates for quantum information processing applications given their excellent coherence times. Designing a scalable donor-based quantum computer will require both knowledge of the relationship between device geometry and electron tunnel couplings, and a spin readout strategy that uses minimal physical space in the device. Here we use radio frequency reflectometry to measure singlet–triplet states of a few-donor Si:P double quantum dot and demonstrate that the exchange energy can be tuned by at least two orders of magnitude, from 20 μeV to 8 meV. We measure dot–lead tunnel rates by analysis of the reflected signal and show that they change from 100 MHz to 22 GHz as the number of electrons on a quantum dot is increased from 1 to 4. These techniques present an approach for characterizing, operating and engineering scalable qubit devices based on donors in silicon.

## Abstract (web summary)

Donor spin states in silicon are good quantum bit candidates due to their long coherence times. Here, the authors use radio frequency reflectometry to measure singlet and triplet states, and to determine the tunnel coupling between few-donor silicon double quantum dots and the electrical leads.

## Graphical abstract

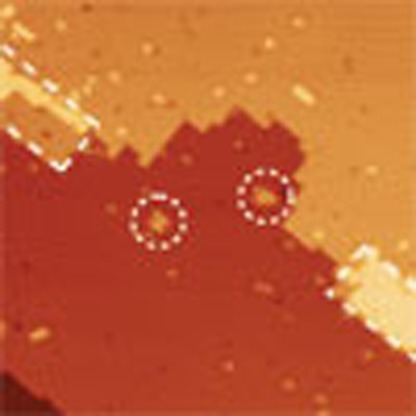

The electron and nuclear spin states of phosphorus donors in silicon are highly promising systems for quantum information processing applications^1^, featuring extremely long coherence times in isotopically enriched ^28^Si (refs 1, 2, 3, 4). In addition to early proposals by Kane^5^ and by Loss and DiVincenzo^6^, several other types of spin qubits hosted on donors, multiple-donor quantum dots^7^,^8^ or electrostatically defined quantum dots have since been proposed, including singlet–triplet qubits^9^, exchange-only qubits^10^ and hybrid qubits^11^. In each case, these strategies for encoding and manipulating quantum information require a controllable exchange interaction between two electrons and rely on Pauli blockade to readout the singlet–triplet state of the electron pairs^8^,^12^. Spin qubits hosted on multi-donor quantum dots offer additional advantages in addressability and in the tunability of the exchange interaction^7^.

A recent trend in measuring quantum dots and donors is the use of radio frequency (RF) reflectometry to detect single-electron tunnelling^13^,^14^,^15^,^16^,^17^,^18^,^19^,^20^. An RF voltage signal applied to a quantum dot drives single-electron tunnelling, an alternating current response that can be measured and expressed as a complex admittance^21^,^22^. When applied to double quantum dots, tunnelling between the dots is allowed for a spin singlet state but not for triplet states, which provides a direct way to distinguish the singlet–triplet spin character of the electrons^13^,^18^. This non-destructive spin measurement has the advantage that the RF probe signal can be introduced via an existing control electrode, so it does not require a separate charge detection structure to be fabricated in the device^23^,^24^. This is particularly advantageous in donor-based qubits where the interdonor separations required to make exchange interactions possible are small (≲20 nm), leaving little room to incorporate control gates and charge detectors in multi-qubit devices. In contrast to larger gate-confined quantum dots, tunnel couplings in donor-confined systems are less influenced by gate voltages *in situ* and must instead be set by the device geometry at the fabrication stage. Therefore, one of the challenges that must be met in designing spin qubit devices based on donors is to understand the physics of electron confinement and tunnelling so that the appropriate couplings for spin detection and manipulation can be engineered into the devices.

In this work, we use RF reflectometry to measure singlet–triplet states and tunnel rates in an atomic precision few-donor silicon double quantum dot device patterned by scanning tunnelling microscope (STM) lithography^8^. By analysing the amplitude and phase of the reflected RF signal, we measure dot–lead tunnel rates and show that they change markedly for each electron added to a dot, from 100 MHz for the first electron added to a dot composed of three P atoms, to 22 GHz for the fourth electron. We identify Pauli spin blockade between the two dots, accurately measure the exchange energy of the singlet–triplet system and demonstrate that it can be tuned by at least two orders of magnitude with in-plane, monolayer-doped gates. We demonstrate readout of the singlet excited state at high magnetic field and measure a singlet–triplet relaxation time (60 ns), which is limited by a co-tunnelling process happening between the dots and leads in this device. These results demonstrate a path towards making scalable spin qubit devices with donors, using RF reflectometry to minimize the device complexity and engineer the tunnel couplings to desirable values.

## Results

### Device fabrication and characterization

**Figure 1:**
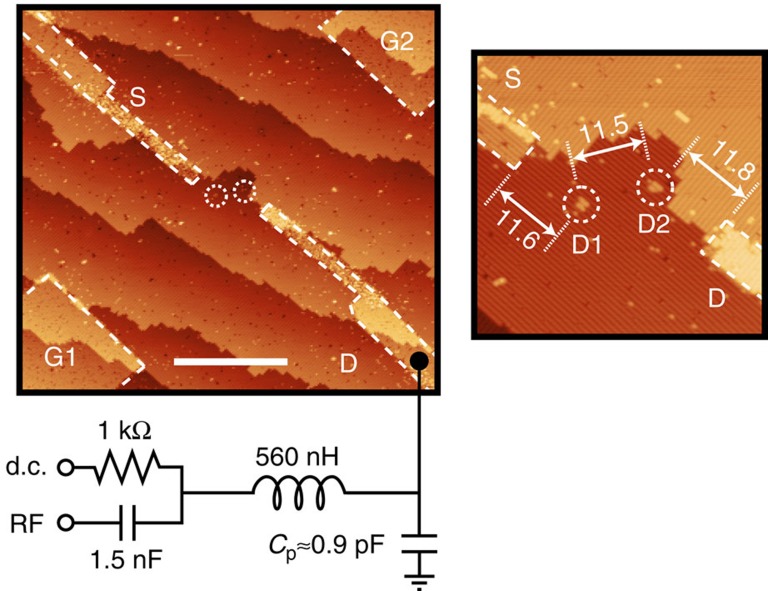
Reflectometry detection of a few-donor double quantum dot. Scanning tunnelling microscope image of two quantum dots, D1 and D2, patterned in a lithographic H mask. The quantum dots are composed of two and three P donors, respectively. Source (S) and drain (D) leads allow current to be passed through the dots for transport measurements. A RF tank circuit is formed by a 560 nH inductor connected to contact D and its parasitic capacitance to ground *C*_p_, as shown. A 1-kΩ resistor and 1.5-nF capacitor form a bias tee that separates the RF port from a d.c. bias port. Scale bar (white), 40 nm. The inset shows a zoom-in image of the double quantum dots, with dimensions in nanometre.

Figure 1 shows an image of the lithographic mask used to define two quantum dots (D1 and D2), composed of a cluster of two P donors and a cluster of three P donors, respectively. Wires are patterned in the same phosphorus monolayer as the dots to form source (S) and drain (D) leads, and two gates (G1 and G2) used to control the electrostatic potentials of the two dots. A tank circuit is connected to the drain, composed of a 560 nH inductor, which together with a parasitic capacitance, *C*_p_≈0.9 pF, forms a resonance at *f*_0_=222.6 MHz with quality factor *Q*≈35.

**Figure 2:**
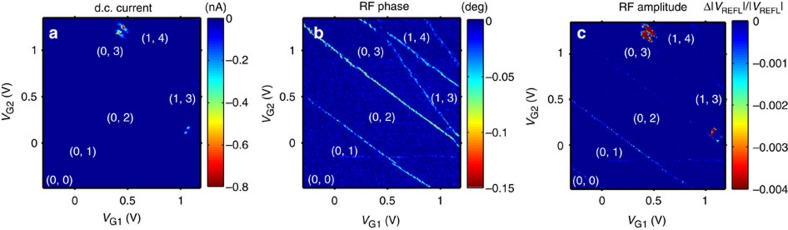
Double quantum dot stability diagram compared in d.c. transport and in RF reflectometry. Stability diagram of the double quantum dot measured in (**a**) d.c. transport current through the two dots, (**b**) relative phase of the reflected RF signal; Δ*φ*, and (**c**) relative RF amplitude, Δ|*V*_REFL_|/|*V*_REFL_|. Charge configurations are labelled (*N*_1_ and *N*_2_) for *N*_1_ electrons on dot 1 and *N*_2_ electrons on dot 2. A source–drain bias *V*_D_=−3 mV was applied.

Figure 2 shows the transport current (Fig. 2a) and changes in the phase (Fig. 2b) and amplitude (Fig. 2c) of the reflected RF signal as a function of the two gate voltages. D.c. current appears only near the triple points where charge transitions of both dots come into resonance with the Fermi levels of the leads and with each other^25^. In contrast, the individual charge transitions on each quantum dot are also clearly visible in the RF measurements because only one resonance condition needs to be met. A.c. can be driven, either between one quantum dot and its lead or between the two quantum dots, even when the d.c. through the two dots in series is blockaded. The RF responses confirm the electron numbers and binding energies estimated previously for this device from d.c. transport data^8^.

### Complex admittance analysis

The admittance *Y*(*ω*) of single-electron tunnelling between a dot and a lead has both a real and imaginary part, represented as *Y*(*ω*)=*g*_q_+*iωC*_q_, a quantum capacitance *C*_q_ in parallel with quantum conductance *g*_q_. This admittance depends on the tunnel rate *γ*, the drive frequency *ω*_0_ and the temperature of the electrons in the lead, *T*_e_ (ref. 22). These two types of admittance can be distinguished in the measurements because adding a dissipative load (conductance) to the resonant circuit absorbs energy and reduces the amplitude of the reflected signal, while a dispersive load (capacitance) shifts the resonant frequency of the circuit, which looks like a phase shift of the reflected signal when measured at a fixed frequency. Interestingly, in Fig. 2b,c, we see that transitions between dots and leads appear primarily in the phase response except at the lower electron numbers, where they appear more strongly in the amplitude channel. These complementary pieces of information allow us to estimate the tunnel rates, providing valuable guidance on how to design donor-based devices so that the tunnel rates are in a range for optimal detection sensitivity and qubit operations.

**Figure 3:**
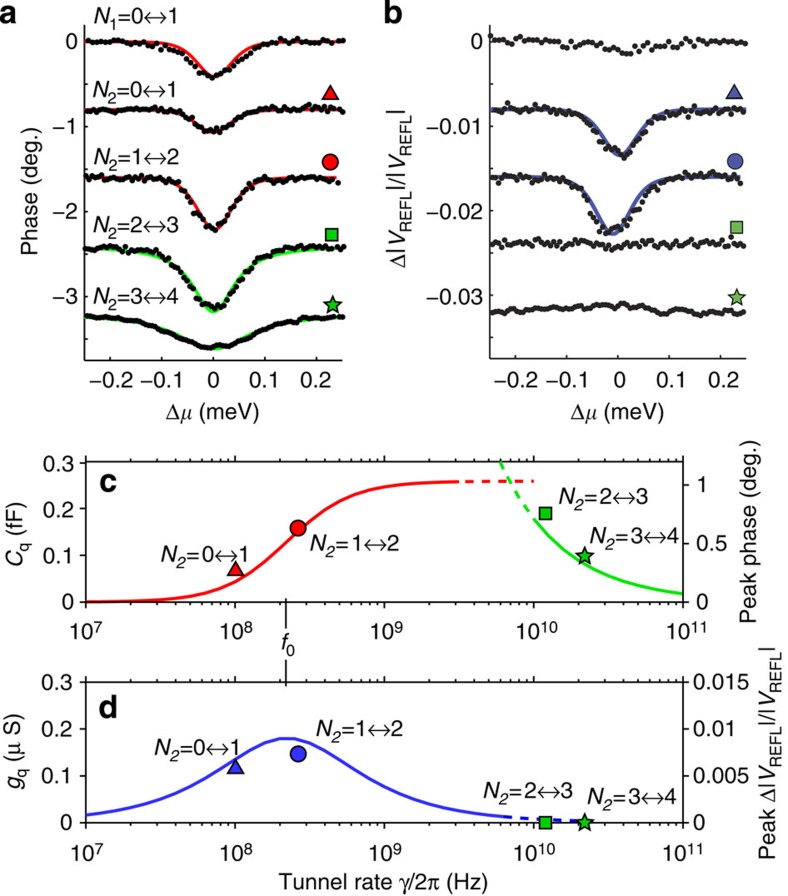
Extraction of tunnel rates at dot–lead transitions. (**a**) Phase response of each electron transition as a function of potential relative to the Fermi level of the lead, Δ*μ*. Red curves are fits to equation (1) for the thermally broadened transitions, green curves are fits to equation (3) for the lifetime-broadened transitions. Curves are offset from one another by 0.75° for clarity. (**b**) Amplitude response of each transition. Significant amplitude responses for *N*_2_=0↔1 and *N*_2_=1↔2 indicate a finite quantum conductance. Blue curves are fits to equation (2). (**c**) Peak quantum capacitance of a dot–lead transition predicted as a function of tunnel rate for 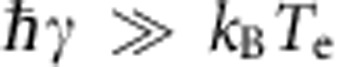 (Equation 3, green curve) and 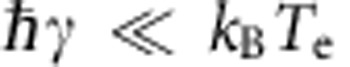 (Equation 1, red curve). The lines are dashed in the intermediate regime 
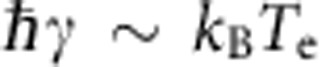
 where neither limit applies. The peak phase shift for each charge transition, scaled to capacitance, is plotted for those transitions where the tunnel rate can be determined from line broadening (green squares), or estimated from the relative peak phase shift (red triangles). (**d**) Quantum conductance *g*_q_ as a function of tunnel rate (Equation 2, blue curve). Blue triangles and green squares indicate the scaled peak amplitude response of each transition at its estimated tunnel rate.

To illustrate this, in Fig. 3, we plot the phase (Fig. 3a) and amplitude (Fig. 3b) responses of each of the dot–lead transitions as a function of the chemical potential of the dot relative to the Fermi level of the lead, Δ*μ*. These traces were taken well away from the triple-point charge degeneracies to avoid any co-tunnelling or transport effects. Three of the transitions, one of dot 1 and the two lowest transitions of dot 2, each have different peak phase and amplitude response, but all three have the same width in Δ*μ*. This indicates that they are in the regime in which the width of the transitions is determined by the electron temperature, 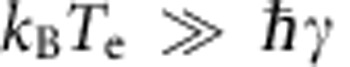. In this regime, the quantum capacitance and conductance are given by^22^,^24^


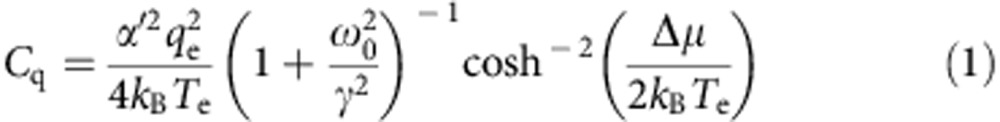



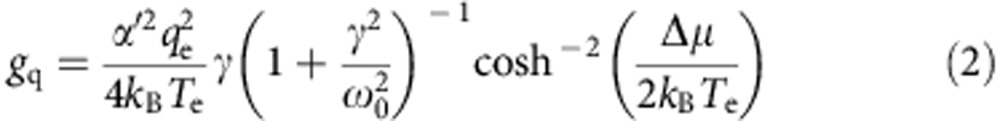


where *q*_e_ is the electronic charge unit and *α*′ is the geometric factor relating the change in the chemical potential of the dot to the Fermi level of the lead. By fitting these first three transitions to the cosh^−2^ part of equation (1) (red curves in Fig. 3a), we determine the electron temperature of the leads *T*_e_=260 mK. The peak quantum capacitance *C*_q_ at Δ*μ*=0 predicted by equation (1) (red curve in Fig. 3c) is zero for tunnel rates small compared with *ω*_0_ because electron tunnelling cannot keep up with the driving force. It then increases to an inflection point at *γ*=*ω*_0_ and levels off to 
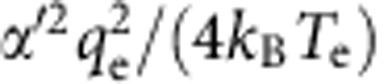
 for 
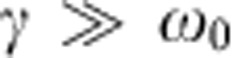
, where the maximum value is limited by the device geometry (through parameter *α*′) and temperature. The peak conductance *g*_q_ given by equation (2) (blue curve in Fig. 3d) is significant only when *γ*∼*ω*_0_. For higher tunnel rates, electron tunnelling happens out of phase with the driving signal, so the response is purely capacitive. Thus, the relative phase and amplitude responses at each transition allow us to estimate the tunnel rate for *γ*∼*ω*_0_. For the dot 1 transition, *N*_1_=0↔1, the lack of an amplitude response indicates that the tunnel rate is much greater than the drive frequency, but it is still less than the thermal energy (as we discuss below) so it is on the order of 1 GHz. The first transition of dot 2, *N*_2_=0↔1, has a reduced phase response but a significant amplitude response, an indication that the tunnel rate is comparable to the drive frequency. The second transition, *N*_2_=1↔2, is similar but with larger phase response. The scaling between amplitude/conductance and phase/capacitance is discussed in Supplementary Note 1. By fitting the relative peak conductance and capacitance responses for each transition to equations (1) and (2), we estimate *γ*/(2*π*)≈100 MHz for *N*_2_=0↔1 and *γ*/(2*π*)≈250 MHz for *N*_2_=1↔2. The peak phase response of these two transitions is plotted against these tunnel rate estimates as a triangle and circle in Fig. 3c, for comparison with equation (1) (red curve) and the peak amplitude responses are plotted in Fig. 3d for comparison with equation (2) (blue curve).

In contrast, the two highest electron number transitions of dot 2, *N*_2_=2↔3 and *N*_2_=3↔4, have responses that are broader than the others in Δ*μ*, an indication that the tunnel rate is large, ℏ*γ*>*k*_B_*T*_e_. In this regime, the electron tunnelling has a purely capacitive response^22^


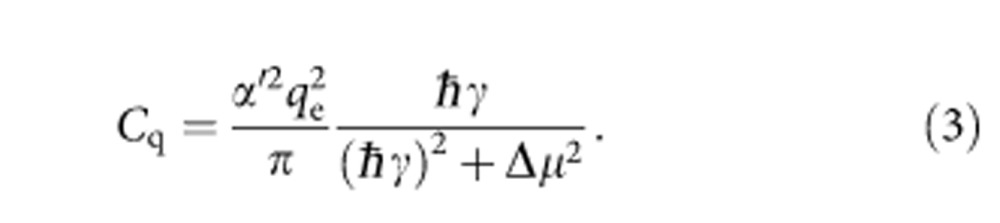


By fitting equation (3) to these two transitions (green curves in Fig. 3a), we estimate tunnel rates *γ*/(2*π*)=22 GHz for *N*_2_=3↔4 and *γ*/(2*π*)=11 GHz for *N*_2_=2↔3. The peak phase response of these two transitions is plotted as a green square and star in Fig. 3c, for comparison with the tunnel rate dependence of equation (3) (green curve).

Taken together, the results from dot 2 demonstrate that tunnel coupling changes markedly for each electron transition, reflecting the unique physics of donor-confined electrons. As electrons are added to the quantum dot the multi-electron wavefunction expands spatially, overlapping more with the lead and giving a larger tunnel coupling. The measurements nicely illustrate the full range of tunnel rates over which reflectometry detection is most favourable. For tunnel rates comparable to the drive frequency (*γ*∼*ω*_0_), the peak response (combined phase and amplitude) is reduced and there is dissipation in the tunnelling, which introduces noise in the measurement^24^. For large tunnel rates, the measured phase shift becomes broader and smaller in magnitude as the quantum dot becomes less well confined. Future donor-based devices should be designed to have tunnel rates in the middle range for optimized RF detection.

### Spin state readout

**Figure 4:**
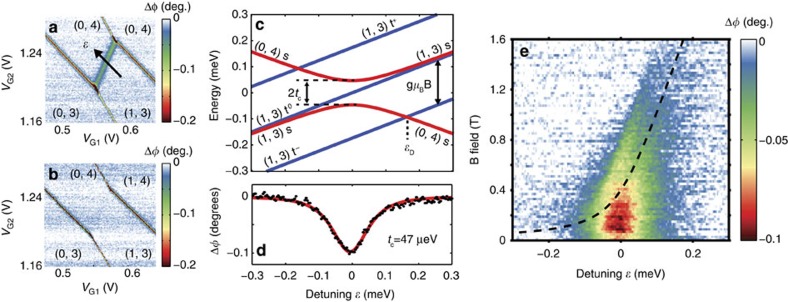
Spin blockade and exchange energy at the (1, 3)–(0, 4) charge transition. (**a**) Stability diagram with *B*=0 T, with interdot tunnelling between the (1, 3) and (0, 4) charge configurations. The arrow indicates the direction of increasing detuning energy *ɛ*. (**b**) Same diagram taken with *B*=2 T. The interdot phase response disappears as the ground state of the system is now a triplet. (**c**) Energy level diagram for the system, including three spin triplet states with (1, 3) charge configuration (blue) and two singlet states (red). With applied magnetic field the ground-state transitions from singlet to triplet at detuning energy *ɛ*_D_. (**d**) Phase response at the interdot transition as a function of detuning, a cut through the data of a along the detuning line. The red curve indicates a fit to equation (4) from which we extract the tunnel coupling *t*_c_=47 μeV. (**e**) Interdot phase measurement as a function of detuning *ɛ* and magnetic field *B*. The phase response disappears asymmetrically as the exchange energy increases for increasing *ɛ*. The dashed line indicates the degeneracy of the lowest energy singlet and triplet states.

In transport measurements, we previously observed Pauli spin blockade at the (1, 3) and (0, 4) charge transition^8^. We now study how the spin blockade influences the RF response. With no applied magnetic field, we observe a phase shift line along the (1, 3) – (0, 4) charge degeneracy as seen in Fig. 4a due to the additional capacitive load presented by the interdot tunnelling. The quantum capacitance at the interdot transition obeys 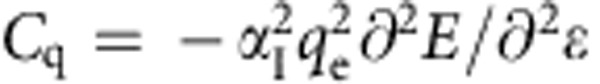, where the detuning energy *ɛ* is the difference in energy between the (0, 4) and (1, 3) charge states, and *α*_I_=*α*_1D_−*α*_2D_=0.32 meV mV^−1^ is the geometric factor that relates *ɛ* to the voltage on lead D, Δ*ɛ*=−*q*_*e*_*α*_I_Δ*V*_D_ (ref. 13). The lowest energy levels of the system near the charge degeneracy line are shown in Fig. 4c (ref. 9). For the lowest energy singlet states, whose energies near the anti-crossing are 
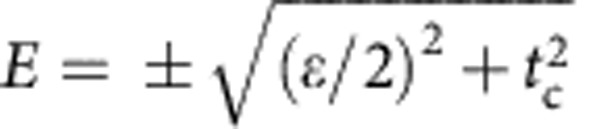
, the quantum capacitance is


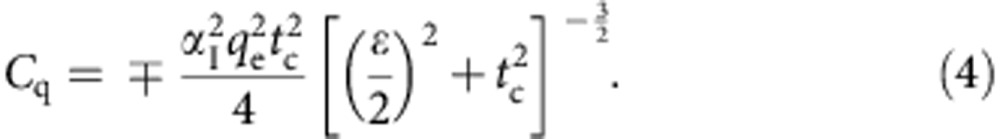


Fitting this function to the width of the interdot transition line as shown in Fig. 4d provides a direct measure of the interdot tunnel coupling *t*_c_=47±5 μeV, improving on the estimate given in ref. 8, which was based on transport data. In Fig. 4b, we repeat the measurement of the interdot transition with a magnetic field *B*=2 T applied, where we see that the interdot phase response disappears completely. Here the magnetic field has lowered the *t*^−^ triplet state in energy so that it is the ground state. Pauli blockade prevents tunnelling when the spin state of the electrons is a triplet so there is no quantum capacitance and no measured phase response in this case.

In Fig. 4e, we show how the interdot phase response varies with respect to detuning energy and applied magnetic field to map out the singlet–triplet ground-state transition, which occurs at a detuning 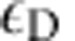 as indicated in Fig. 4c. The phase response disappears at high *B* as the ground state of the system becomes the *t*^−^ triplet state. The dashed line on the plot indicates the singlet–triplet degeneracy point 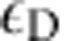 expected given the value of *t*_c_ extracted from Fig. 4d and magnetic *g*-factor *g*=2. We see that the disappearance of the phase response matches well with the expected dependence on *B* and 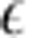. The response line is asymmetric with respect to detuning, which we expect because the exchange energy (energy separation between the *s* and *t*^0^ states) is smaller when the electrons are separated in the (1, 3) configuration and increases as the detuning field pushes them towards the (0, 4) transition. The Zeeman energy at which the phase response disappears at each detuning point constitutes a direct measurement of the exchange energy across the charge degeneracy line, which changes from about 20 μeV at 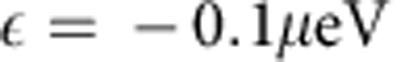 to 120 μeV at 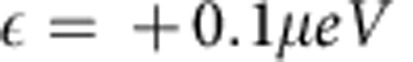. Evidence of larger exchange energies can be seen at higher magnetic fields (Supplementary Note 2). In transport data, we previously observed that the exchange energy is as high as 8 meV at very high detuning^8^, demonstrating that the exchange energy can be tuned by at least two orders of magnitude in donor-based quantum dots using monolayer-doped, in-plane gates. The ground-state transition is not perfectly sharp, consistent with a temperature 120 mK or less (Supplementary Note 3). We note this is certainly less than the electron temperature we measured in the leads, *T*_e_=260 mK; the quantum dot electrons, when tuned away from the Fermi level of the leads, are isolated from thermal fluctuations in the leads and can equilibrate to a lower temperature.

**Figure 5:**
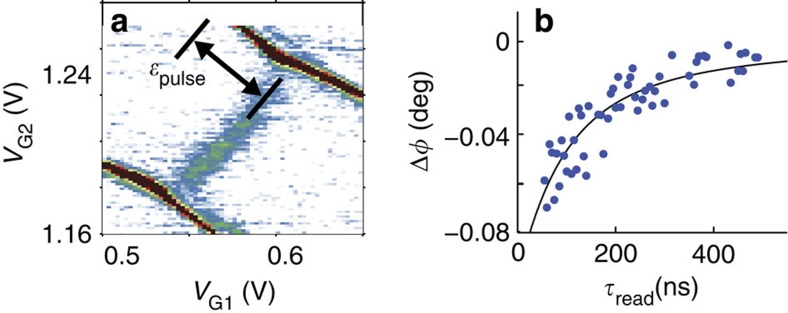
Singlet excited state measurement and relaxation time. (**a**) With *B*=2 T, a detuning pulse of amplitude *ɛ*_pulse_ that populates the singlet state causes the interdot phase response to reappear. (**b**) Magnitude of the phase response as the readout time is varied (blue dots). The black line is a fit to a time-averaged exponential decay, showing that the excited state singlet population decays with a characteristic time 60 ns.

To observe non-equilibrium dynamics of the singlet–triplet system, we apply a continuous train of 10 ns voltage pulses to the gates equivalent to *ɛ*_pulse_=+1 meV, separated by a read time 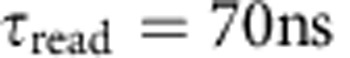, while *B*=2 T. Because *ɛ*_pulse_>*ɛ*_D_, the singlet state becomes the ground state during the pulse and the system may relax to the singlet state during this time. This singlet can then be detected at *ɛ*=0 between pulses. Figure 5a shows the result: the interdot phase response, absent without the pulses as in Fig. 4b, now reappears because the singlet state is populated during the read time. The magnitude of the phase response as a function of the read time is shown in Fig. 5b, which follows the time average of an exponential decay with characteristic time 60 ns. This apparently short relaxation time is not fundamental to the singlet–triplet spin system but related to co-tunnelling of electrons occurring between the quantum dots and the leads in this particular device (Supplementary Note 4). The tunnel coupling between the dots and the leads in this device (*γ*=22 GHz for *N*_2_=3↔4) is too strong to allow for well-isolated double quantum dots, highlighting the value of tunnel rate measurements for the design of donor-based qubit devices. Future devices will address this with weaker coupling to the leads for more robust singlet–triplet spin qubit states.

## Discussion

In summary, using RF reflectometry, we have shown how we can extract the tunnel rates between donor-based quantum dots and their leads and found that these change from 100 MHz to 22 GHz as we increase the number of electrons from 1 to 4 on one of the quantum dots. Using the same technique, we also extracted an accurate measure of the tunnel coupling of 47±5 μeV between the two-P and three-P dots separated by 11.5 nm. The tunnel rate measurements we present here are improvements in accuracy over the estimates we previously made based on transport data^8^, because with RF reflectometry each of the three tunnel barriers in the double quantum dot system can be probed individually. We observed that the exchange coupling at this transition is tunable by two orders of magnitude or more using monolayer, in-plane gates. We found the singlet–triplet relaxation time at the (1, 3)–(0, 4) transition to be ∼60 ns. This fast relaxation rate is not inherent to the spin system but significantly affected by the strong tunnel coupling (∼20 GHz) to electronic states in the leads. These results demonstrate the advantages of RF detection for singlet–triplet readout, measurement of tunnel coupling and exchange coupling in donor-based devices, and represent important steps towards implementing the Kane model of quantum computation using phosphorus nuclear spins in silicon^5^.

## Methods

### Device fabrication

The phosphorus doping profile is patterned by STM lithography in an ultrahigh vacuum chamber. The Si sample (001) surface is prepared in the 2 × 1 reconstruction by a flash anneal to 1,150 °C, and then hydrogen passivated by introducing atomic hydrogen into the vacuum chamber. Controlled voltage and current pulses on the STM tip locally desorb the hydrogen mask to define the device features. PH_3_ gas introduced into the chamber binds to the surface in the regions where the hydrogen was desorbed. An anneal to 350 °C causes the P atoms to incorporate into the Si crystal. The P features are encapsulated by solid source molecular beam epitaxy of 25 nm of Si, grown at 250 °C. The doped leads are contacted by first using reactive ion etching to make holes in the encapsulation layer down to the doped layer, and then the holes filled in by evaporation of Al. The resulting contact between Al leads and the P-doped monolayer is ohmic. The sample was mounted on a custom-printed circuit board, which included the inductor for a resonant circuit and bias tee resistors and capacitors.

### Experimental set-up

All measurements are performed in a dilution refrigerator operating at 42 mK unless otherwise specified. The RF drive signal, ≈−90 dBm power, is introduced through the RF port (Fig. 1), the reflected signal is routed by a directional coupler towards an RF amplifier (Minicircuits ZX60-P103-LN+) mounted at the 4 K stage of the refrigerator and then further amplified at room temperature before being homodyne quadrature demodulated to extract the amplitude and phase of the reflected signal^20^.

## Additional information

**How to cite this article:** House, M. G. *et al*. Radio frequency measurements of tunnel couplings and singlet–triplet spin states in Si:P quantum dots. *Nat. Commun.* 6:8848 doi: 10.1038/ncomms9848 (2015).

## Supplementary Material

Supplementary Information Supplementary Figures 1-5, Supplementary Notes 1 -4 and Supplementary References.
