# Supplementary material for: Radio frequency measurements of tunnel couplings and singlet–triplet spin states in Si:P quantum dots
Source: Nat Commun. 2015 Nov 9;6:8848. doi: 10.1038/ncomms9848 (PMC4667619; doi:10.1038/ncomms9848)
Supplement: Supplementary Information — Supplementary Figures 1-5, Supplementary Notes 1 -4 and Supplementary References. [file ncomms9848-s1.pdf]

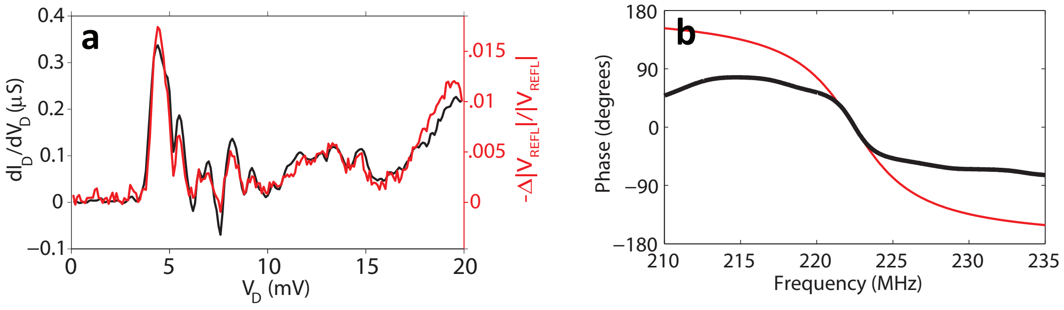

Supplementary Figure 1. **Calibrating admittance measurements.** (**a**) Comparison between the dc conductance measured at a triple point (black, left hand scale) and the fractional reflected signal amplitude (red, right hand scale) as a function of drain bias voltage. The scaling factor which relates the two is $-2.0 \cdot 10^{-5}\,\mu$S. (**b**) Phase of the reflected signal as a function of frequency near the resonance frequency 222.6 MHz. The slope of the phase with respect to frequency (black curve) is used to estimate the quality factor of the circuit $Q \approx 35$ from a lumped-element circuit model (red curve). This quality factor and the total capacitance of the circuit are used to establish the relationship between phase response and quantum capacitance.

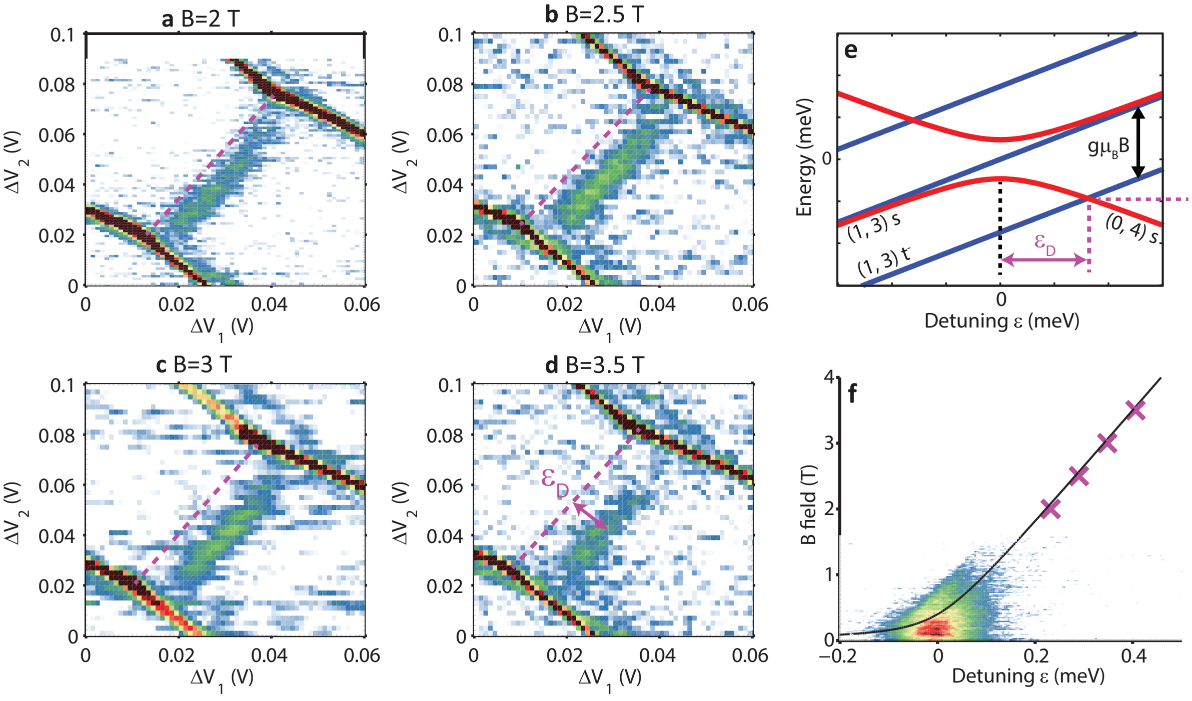

Supplementary Figure 2. **Singlet excited state observations at various magnetic fields.** Four panels show the observation of singlet states at (**a**) B=2.0 T, (**b**) B=2.5 T, (**c**) B=3.0 T, and (**d**) B=3.5 T during the application of a 10 ns / 70 ns pulse train along the detuning axis of amplitude $\Delta\epsilon = 1$ meV. The phase response which indicates singlet states is offset from the line connecting the triple points (magenta dashed line). (**e**) Energy level diagram of the singlet-triplet system, highlighting that while the singlet (1, 3) - (0, 4) anti-crossing occurs at $\epsilon = 0$, at high magnetic field the ground state transition between (1, 3) and (0, 4) charge configurations occurs at ϵ_{D}. (**f**) Fig. 4e from the main text is reproduced, with four data points added which indicate ϵ_{D} measured from the distance between the singlet response line and the line connecting the triple points in (**a**)-(**d**) (magenta crosses). These show good agreement with the expected dependence of the exchange energy on ϵ (black line).

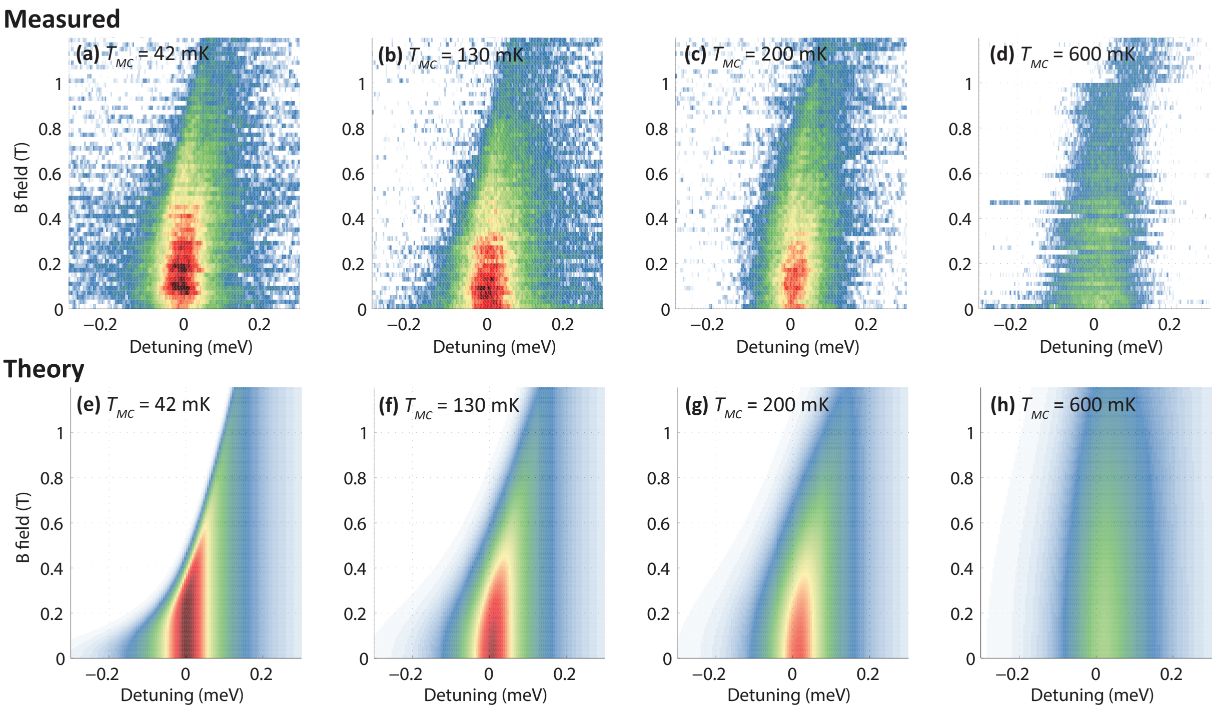

Supplementary Figure 3. **Temperature dependence of Pauli blockade measurements.** The rf phase response at the (1, 3) - (0, 4) charge transition as a function of detuning ϵ and magnetic field B, are shown at four different mixing chamber temperatures, (**a**) $T_{\rm MC} = 42\,$mK, (**b**) $T_{\rm MC} = 130\,$mK, (**c**) $T_{\rm MC} = 200\,$mK, (**d**) $T_{\rm MC} = 600\,$mK. Shown for comparison in (**e**)-(**h**) are corresponding simulations of the signal, assuming a simple model with the temperature of the singlet-triplet system equal to the mixing chamber temperature. At $T_{\rm MC} = 42\,$mK we see that the disappearance of the phase response with increasing B happens more smoothly than predicted by the model, suggesting the singlet-triplet system is at a higher temperature than the mixing chamber. At temperatures $T_{\rm MC} = 130\,$mK and above, there is good agreement between the measurements and the model predictions.

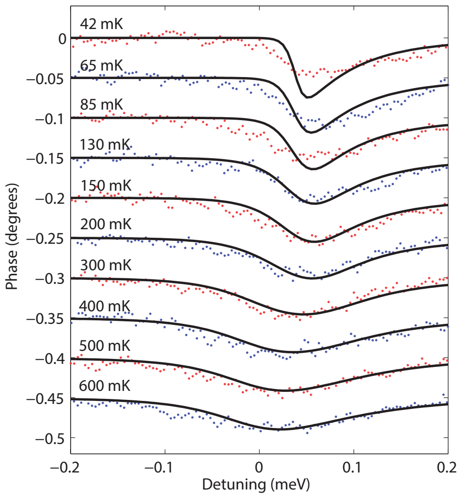

Supplementary Figure 4. **Temperature dependence of the singlet-triplet ground state transition at B=0.6 T.** The inter-dot phase response is shown at various temperatures of the mixing chamber. Solid lines indicate theoretical predictions as discussed in the text. At low mixing chamber temperature there is disagreement between the observed line shape and the model, but it is a good fit at 130 mK and above. Each curve is offset by 0.05 degrees for clarity.

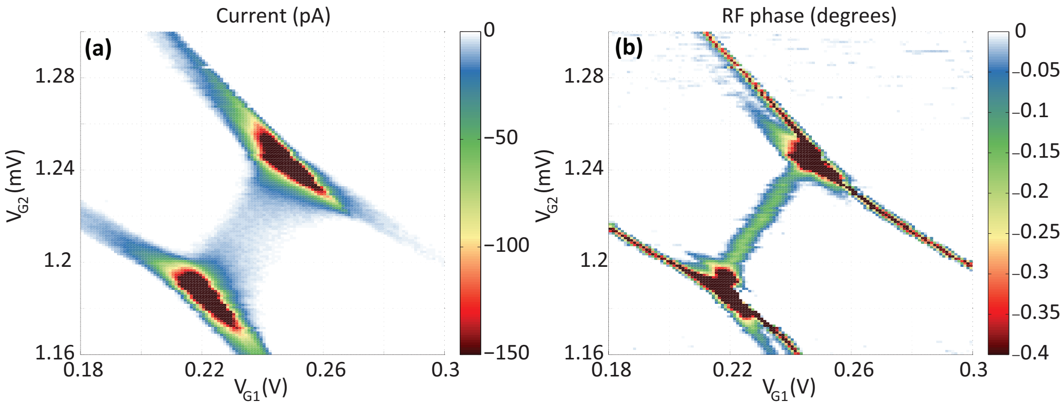

Supplementary Figure 5. **Co-tunneling current observed at the (1,3)-(0,4) transition.** (**a**) Current and (**b**) rf phase response (**b**) at the (1,3)-(0,4) transition with B=2 T and bias $V_{\mathrm{D}} = -1$ mV. Current flows within the bias triangles, as expected, but also a small current flows along the inter-dot transition line. This indicates a co-tunneling process by which a virtual, simultaneous tunneling of two electrons allows current to flow. This current allows the singlet state to be populated, as seen by the appearance of the inter-dot transition phase response in (**b**). This is clear evidence that in this device the double dot system and its spin states are not well isolated from the electronic states of the leads.

SUPPLEMENTARY NOTE 1: RELATING REFLECTED SIGNAL RESPONSE TO DEVICE ADMITTANCE

We experimentally determine the relationship between the change in the reflected signal amplitude and the conductance (real admittance) used in Fig. 3d of the main text by comparing the reflected amplitude response with dc current measurements. Such a comparison is shown in Fig. 1a. The dc conductance $dI_{\mathrm{D}}/dV_{\mathrm{D}}$ measured while varying the drain voltage near a triple point is shown in black and plotted on the left hand scale. It has significant structure due to excited states of the two dots and density of states fluctuations in the leads. The rf amplitude response measured simultaneously is plotted in red against the right hand scale. The two data sets were fit to a linear relationship to determine the scaling factor, $-2.0 \cdot 10^{-5}\,\mu$S per unit of $\Delta|V_{\mathrm{REFL}}|/|V_{\mathrm{REFL}}|$. The amplitude response is nonlinear for large conductances, but that is not a significant effect for the range of g_{q} we measure.

We determine the scaling between reflected phase response and quantum capacitance by comparing to the frequency response of the resonator, which is shown in Fig. 1b. The black curve shows the measured phase as a function of rf frequency across the resonance. The red curve shows the fit of the phase response to a simple model of a resonance with input impedance $Z_{\mathrm{IN}}(f) = R(1 + 2iQ(f - f_0)/f_0)$, where R is the internal resistance of the resonator. Losses caused by R were dominated by losses due to the coupling with the external transmission line. In this (overcoupled) regime, $R \ll Z_0$, the reflection coefficient $\Gamma = (Z_{\mathrm{IN}} - Z_0)/(Z_{IN} + Z_0)$ has phase

$$\phi(f) = \arg(\Gamma) = \arctan\left[\frac{4Q\left(\frac{f-f_0}{f_0}\right)}{4Q^2\left(\frac{f-f_0}{f_0}\right)^2 - 1}\right] \tag{1}$$

where $Q = \omega_0 L/Z_0$. We estimate $Q \approx 35$ from the slope of the phase at the resonant frequency, $d\phi/df = -4Q/f_0$. The red curve in Fig. 5b shows Eq. 1 for this value of Q. The measured phase deviates from the red curve away from the resonance because of the finite impedance of the 1 kΩ bias tee resistor. When a small (quantum) capacitance is added to this circuit the resonant frequency $f_0 = (2\pi\sqrt{LC})^{-1}$ shifts by $df_0/dC = -f_0/(2C)$, so the expected phase response $d\phi/dC$ is $-2Q/C$, which evaluates to $2.3 \cdot 10^{-16}$ F deg^{-1} for the parameters of this experiment. This relationship was used to scale between phase response and capacitance in Fig. 3c in the main text.

SUPPLEMENTARY NOTE 2: PULSED-GATE SINGLET EXCITED STATE OBSERVATIONS

In Fig. 2 we show excited singlet state observations, at high magnetic fields (a) B =2 T, (b) B =2.5 T, (c) $B = 3$ T, and (d) $B = 3.5$ T while applying a pulse to excite the system as described in the main text. We see that as we increase B the singlet charge degeneracy line where the phase response occurs moves away from the (1, 3) - (0, 4) charge degeneracy line connecting the triple points (magenta dashed line in the figures). The triple points occur where the lowest energy (1, 3) charge state, (0, 4) charge state, and the Fermi level of the leads E_{F} are all equal in potential. When $B = 0$ T this happens at $\epsilon = 0$ where the (1, 3) - (0, 4) singlet anti-crossing is centered, but when $g\mu_{\mathrm{B}}B > t_{\mathrm{c}}$ the lowest energy charge degeneracy is between the (1, 3)t^- state and the (0, 4)s state, which occurs at detuning ϵ_{D}, as shown in Fig. 2(e). Thus the observed distance between the singlet degeneracy line and the line connecting the triple points is another measure of the exchange energy. In Fig. 2(f) we show the data from Fig. 4(e) in the main text, rescaled to view the extended prediction of the exchange energy to higher ϵ and B (black line). Four crosses mark the four measurements of ϵ_{D} taken from the distance between two lines in Fig. 2(a)-(d). We see there is very good agreement with the expected exchange energy, extending our observation of the tunability of the exchange energy to $> 400\,\mu$eV.

SUPPLEMENTARY NOTE 3: DETERMINING THE TEMPERATURE OF THE SINGLET-TRIPLET SYSTEM

One useful aspect of singlet-triplet spin readout is that it involves only electronic states which are well isolated from the thermal populations of the leads, unlike single-spin readout by energy selective tunneling [1]. This can be a significant advantage, as it is an experimental challenge to thermalize the leads at millikelvin temperatures; indeed in this experiment the electron temperature of the leads was $T_{\mathrm{e}} = 260$ mK (see main text) while the mixing chamber base temperature was 42 mK. Here we observe directly from the reflectometry measurements that the double quantum dot singlet-triplet system is isolated from the thermal fluctuations of the leads and its temperature is lower than T_{e}.

Supplementary Figure 3(a) shows the phase response of the (1, 3) - (0, 4) charge transition, which disappears at high magnetic field as the ground state of the system becomes a triplet as discussed in the main text. The disappearance of the phase response with increasing B is not perfectly sharp, which could be due to finite thermal populations of the two lowest energy states near this transition, voltage noise on the gates, or charge fluctuation noise originating in the device itself. Supplementary Figure 3(e) shows the phase response predicted by a model based on a partition function population of the singlet and triplet states [2], using the assumption that the temperature of the singlet-triplet system is equal to $T_{\mathrm{MC}} = 42\,\mathrm{mK}$. This model predicts a sharper cutoff of the phase response than actually observed, meaning that either the effective temperature of the singlet-triplet system is higher than 42 mK or that the resolution of the measurement is limited by voltage or charge noise. Supplementary Figure 3(b) shows the same measurement taken at $T_{\mathrm{MC}} = 130\,\mathrm{mK}$, which looks substantially the same as Supplementary Figure 3(a), but is now in good agreement with the thermal model prediction for $T_{\mathrm{MC}} = 130\,\mathrm{mK}$, Supplementary Figure 3(f). At the higher temperatures, Supplementary Figure 3(c) and (d), the overall magnitude of the signal drops as the triplet states (which contribute no phase shift) and the first excited singlet state (which contributes an opposite phase shift) become thermally populated near $\epsilon = 0$ even for small B. These are in good agreement with the corresponding theoretical predictions in Supplementary Figure 3(g) and (h).

The difference between the experimental results and the model predictions is clearer in Supplementary Figure 4, in which the detuning line profile is plotted for B=0.6 T, at various mixing chamber temperatures. The solid black line is the predicted response for an equilibrium population at each temperature. There is good agreement in the line shape for 130 mK and above, so the observations are consistent with the singlet-triplet system being at a temperature less than 130 mK. From the shape of the response at 42 mK we estimate an upper bound on the temperature of 120 mK. Interestingly we see that the temperature of the singlet-triplet system is less than the electronic temperature of the leads, $T_{\mathrm{e}} = 260\,\mathrm{mK}$, which shows that it is possible to isolate a double quantum dot system from thermal fluctuations of the leads and achieve a lower temperature. This might be relevant for initialization of singlet-triplet qubits by relaxation to the ground state, for example if the exchange energy in a particular device cannot be tuned to be larger than the thermal energy of the leads.

We do not observe a temperature-dependent increase in the signal at the singlet-triplet degeneracy as predicted in Ref. [3]. The discrepancy may be because that model implicitly assumed the singlet-triplet states thermalize on a timescale much faster than the rf drive period, which is not the case in the present experiment.

SUPPLEMENTARY NOTE 4: REASON FOR SHORT T_1: ELECTRON EXCHANGE WITH THE LEADS

In pulsed measurements we observe the excited singlet state at high magnetic field and it has an unexpectedly short relaxation time, $\sim 60\,\mathrm{ns}$, which we found to be independent of B. This relaxation is caused by a virtual exchange of electrons with the lead, in which one electron tunnels out of the dots with another electron simultaneously tunneling in. The new electron can bring with it a different spin state, which favors relaxation to the ground state. Evidence for this process can be seen by applying a small bias, $V_{\mathrm{D}} = -1\,\mathrm{mV}$, along with $B = 2\,\mathrm{T}$ as shown in Supplementary Figure 5 (no pulse is applied). This bias is smaller than the mutual charging energy of the two dots (5.75 meV), so the bias triangles should be well separated, with no current between them. However, we see in Supplementary Figure 5(a) that current does flow along the inter-dot charge transition via a virtual co-tunneling process. This current was present with or without the rf drive signal on. At the same time in Supplementary Figure 5(b) a phase response appears in the rf signal along the inter-dot transition line, indicating significant population of the singlet state. Transport through singlet states is happening even though this process is not allowed classically by energy considerations. The effect of the co-tunneling is present even when a bias is not applied, which is why the spin relaxation time is limited to 60 ns and is independent of magnetic field. Such virtual processes are second-order with respect to the tunnel couplings between the dots and the leads, therefore future devices can be designed with weaker coupling between the dots and the leads in order to suppress this effect.

SUPPLEMENTARY REFERENCES

[1] J. M. Elzerman, R. Hanson, L. H. W. van Beveren, L. M. K. Vandersypen, and L. P. Kouwenhoven, Appl. Phys. Lett. **84**, 4617 (2004).
[2] M. D. Schroer, M. Jung, K. D. Petersson, and J. R. Petta, Phys. Rev. Lett. **109**, 166804 (2012).
[3] A. Cottet, C. Mora, and T. Kontos, Phys. Rev. B **83**, 121311(R) (2011).
